# Analysis of microRNA profile of *Anopheles sinensis* by deep sequencing and bioinformatic approaches

**DOI:** 10.1186/s13071-018-2734-7

**Published:** 2018-03-12

**Authors:** Xinyu Feng, Xiaojian Zhou, Shuisen Zhou, Jingwen Wang, Wei Hu

**Affiliations:** 1 National Institute of Parasitic Diseases, Chinese Center for Disease Control and Prevention, Key Laboratory of Parasite and Vector Biology, National Health and Family Planning Commission, WHO Collaborating Center for Tropical Diseases, National Center for International Research on Tropical Diseases, Shanghai, 200025 People’s Republic of China; 2 Joint Research Laboratory of Genetics and Ecology on Parasites-hosts Interaction, National Institute of Parasitic Diseases - Fudan University, Shanghai, 200025 China; 3 Institute of Software Engineering, Zhejiang University, Hangzhou, 310011 China; 4 State Key Laboratory of Genetic Engineering, Ministry of Education Key Laboratory of Contemporary Anthropology, Collaborative Innovation Center for Genetics and Development, School of Life Sciences, Fudan University, Shanghai, 200438 People’s Republic of China

**Keywords:** microRNA, Next-generation sequencing, Bioinformatic approach, *Anopheles sinensis*

## Abstract

**Background:**

microRNAs (miRNAs) are small non-coding RNAs widely identified in many mosquitoes. They are reported to play important roles in development, differentiation and innate immunity. However, miRNAs in *Anopheles sinensis*, one of the Chinese malaria mosquitoes, remain largely unknown.

**Methods:**

We investigated the global miRNA expression profile of *An. sinensis* using Illumina Hiseq 2000 sequencing. Meanwhile, we applied a bioinformatic approach to identify potential miRNAs in *An. sinensis*. The identified miRNA profiles were compared and analyzed by two approaches. The selected miRNAs from the sequencing result and the bioinformatic approach were confirmed with qRT-PCR. Moreover, target prediction, GO annotation and pathway analysis were carried out to understand the role of miRNAs in *An. sinensis*.

**Results:**

We identified 49 conserved miRNAs and 12 novel miRNAs by next-generation high-throughput sequencing technology. In contrast, 43 miRNAs were predicted by the bioinformatic approach, of which two were assigned as novel. Comparative analysis of miRNA profiles by two approaches showed that 21 miRNAs were shared between them. Twelve novel miRNAs did not match any known miRNAs of any organism, indicating that they are possibly species-specific. Forty miRNAs were found in many mosquito species, indicating that these miRNAs are evolutionally conserved and may have critical roles in the process of life. Both the selected known and novel miRNAs (asi-miR-281, asi-miR-184, asi-miR-14, asi-miR-nov5, asi-miR-nov4, asi-miR-9383, and asi-miR-2a) could be detected by quantitative real-time PCR (qRT-PCR) in the sequenced sample, and the expression patterns of these miRNAs measured by qRT-PCR were in concordance with the original miRNA sequencing data. The predicted targets for the known and the novel miRNAs covered many important biological roles and pathways indicating the diversity of miRNA functions. We also found 21 conserved miRNAs and eight counterparts of target immune pathway genes in *An. sinensis* based on the analysis of *An. gambiae*.

**Conclusions:**

Our results provide the first lead to the elucidation of the miRNA profile in *An. sinensis*. Unveiling the roles of mosquito miRNAs will undoubtedly lead to a better understanding of mosquito biology and mosquito-pathogen interactions. This work lays the foundation for the further functional study of *An. sinensis* miRNAs and will facilitate their application in vector control.

**Electronic supplementary material:**

The online version of this article (10.1186/s13071-018-2734-7) contains supplementary material, which is available to authorized users.

## Background

Malaria is a life-threatening infectious disease caused by the *Plasmodium* spp. parasites and transmitted to individuals through the bites of infected *Anopheles* mosquitoes. Although the global epidemiology of malaria has changed dramatically, still 198 million malaria cases occurred, leading to 584,000 deaths in 2013 [1]. In China, a total number of 3078 malaria cases were reported in 2014, among them, vivax malaria accounting for 89.29% of the indigenous malaria cases [2]. Currently, China is implementing a National Malaria Elimination Program. However, indigenous malaria in some places, together with the great challenge posed by imported *Plasmodium falciparum* malaria [3], would definitely hinder the process of elimination in the coming years.

*Anopheles sinensis*, one of the important malaria vectors in China, is still considered as the primary vector of *P. vivax* malaria due to its wide distribution and high density. *Anopheles sinensis* is a facultative mosquito species, which can take blood meals both from humans and animals but is preferably zoophilic according to a previous ecological study [4]. Malaria used to be prevalent in most parts of the flatlands in China between 25 and 33°N where this species was incriminated as the most dominant malaria vector [5]. According to the recent vector surveillance data, the dominant status of the mosquito has not undergone any fundamental changes, although the natural breeding site environment has changed significantly [6]. To make matters worse, a globally warmer climate [4] and the emerging resistance to a variety of insecticides [7] would together blur the prospect of malaria elimination in China. Effective integrated vector control research is needed to sustain the gains achieved so far and to achieve global malaria elimination in the future.

miRNAs are single-stranded and small non-coding RNAs that play significant roles in the control of gene expression by regulating the expression of target genes at a post-transcriptional level [8]. Through their complicated gene regulatory networks with other non-coding RNAs [9], miRNAs have important functions in most fundamental biological processes including apoptosis, proliferation, differentiation, development, cell cycle control and metabolism [10]. Since the discovery of the first miRNA in *Caenorhabditis elegans* in 1994 by the joint efforts of Victor Ambros’s and Gary Ruvkun’s laboratories [11, 12], thousands of miRNAs have been identified in various organisms, such as mammals, nematodes, and plants, insects and even viruses which were generally believed could not encode miRNAs due to nucleus inaccessibility in the past.

Recently, the miRNA profiles of several mosquito species (*Anopheles gambiae*, *Anopheles stephensi*, *Aedes aegypti*, *Culex fatigans* and *Aedes albopictus* among others) have been successfully identified and characterized [13–16]. Several studies suggested that miRNAs are dysregulated and could play important roles at the post-transcriptional level in physiology, pathogenesis, and immunology. Some of the miRNAs have been frequently demonstrated to be functionally involved in the blood meal, parasite infection or virus infection, such as members of the miR-275 [17], miR-305 [18, 19] and miR-34 [20], indicating that these dysregulated miRNAs may play pivotal roles in the interplay between host and pathogen. However, although *An. sinensis* is an important vector in China, its miRNAs profile and the roles of miRNAs remain largely unfulfilled.

In this study, we performed a comprehensive profiling of miRNAs by high throughput small RNA sequencing and an alternative bioinformatic approach to investigate the miRNAs in *An. sinensis*. Comparisons were made between two approaches to assess the discrimination and difference of identified miRNA profiles. In addition, candidate miRNAs (both conserved and novel miRNAs) validated by two methods were subject to miRNA-gene network analysis to predict the targets of these miRNAs. Furthermore, we investigate the potential roles of miRNAs in *An. sinensis.*

## Methods

### Mosquito rearing and sample preparation

*Anopheles sinensis* (China strain) mosquitoes were reared under insectary in screened cages at 28 ± 2 °C, 70–75% humidity. Adult mosquitoes were provided with constant access to sterile glucose solution and water soaked sponges. After eclosion, adult female samples were pooled with 250 μl of TRIzol® reagent (Invitrogen, California, USA) immediately following collection and stored at -80 °C until further processing. Total RNA was extracted from samples for miRNAs (Takara, Dalian, China) followed manufacturer’s protocol. Quality and purity of RNA were assessed by electrophoresis and ultraviolet spectrophotometry, together with the ratio of OD260 and OD280, of which the value of all samples ranged from 1.8 to 2.2. RNA integrity assess used the Agilent2100 bioanalyzer (Agilent Technologies, Santa Clara, CA, USA). The RIN (RNA integrity number) value of all samples ranged from 8.1–8.9 (scale 1–10), indicating high-quality RNA.

### Small RNA library construction and Illumina sequencing

Small RNAs (sRNAs) libraries were made as manufacturer’s instructions (Illumina Inc, San Diego, USA). sRNAs were isolated by separating total RNAs on denaturing polyacrylamide gel electrophoresis and cutting a portion of the gel corresponding to the size 18–30 nucleotides. The RNAs were ligated with 3' and 5' RNA adaptors. The adaptor-ligated sRNAs were subjected to RT-PCR with 15 cycles of PCR amplification. After gel purification of amplified cDNAs, approximately 20 μg of the pooled RNA samples were submitted for sequencing (Shanghai OE Biotech Co., Shanghai, China) using the Illumina HiSeq 2000.

### Analysis of small RNA sequencing data

First, the initial reads obtained from Illumina sequencing were pretreated to remove adaptors, poly A reads, reads without the insert fragment, and reads of less than 10 nt. Next, the remaining sequences (clean reads) were mapped to the *An. sinensis* genome (GenBank assembly accession: GCA_000441895.2) using SOAP [21] with a tolerance of one mismatch to analyze their distribution. The matched sequences were then queried against GenBank miRBase [22], Rfam [23], and repeats database [24] to annotate the sRNA (rRNAs, tRNAs, snRNAs, snoRNA, miRNA) sequences. Reads overlapping with the exons of protein-coding genes were also excluded to avoid mRNAs contamination. The remnant reads were compared to the miRBase database to identify the conserved miRNAs. Filtered unmatched clean reads were used for miRNA prediction to identify novel miRNAs. The novel miRNAs candidates were predicted by MiRDeep [25] only if they met the criteria described by Allen et al. [26] and Friedlander et al. [27]. The secondary structure, the Dicer cleavage site and the minimum free energy of the unannotated small RNA reads, which could be mapped to *An. sinensis* genome sequences were predicted by using RNA-fold (http://rna.tbi.univie.ac.at/).

### Bioinformatic approach for identification of *An. sinensis* miRNA

We used transcriptome analysis method to identify miRNAs from *An. sinensis*. A total of 3119 stem-loop pre-miRNA sequences belonging to 26 organisms of the subphylum Hexapoda were extracted from miRBase v.21 [22]. RNAseq transcripts of *An. sinensis* deposited in Vectorbase were also downloaded. Pre-miRNAs were used as the query for the search of its homolog in *An. sinensis* transcriptome by the BLAST2.2.22+ program at as described by Krishnan et al- [28] with all parameters as default. The FASTA formats of all the candidate sequences were screened and saved. Prediction and validation of the indentured secondary structures were performed using the M-Fold tool [29] based on thermodynamics metrics. The annotation of mature miRNA was performed based on the widely accepted criteria for selecting miRNAs from pre-miRNA sequences as previously described [30]. Nomenclature of predicted miRNAs was assigned as the pattern of miRBase and prefixed with “asi” to denote *An. sinensis*. The mature sequences were designated “miR” and the precursor hairpins were labeled as “mir”.

### miRNA expression validated by quantitative qRT-PCR

To validate the presence and expression of the identified miRNAs, eight miRNAs with differential expression were selected for qRT-PCR as described previously [31]. The total RNA was extracted using TRIzol Reagent (Invitrogen). The reverse transcription reaction was performed with RevertAid First Strand cDNA Synthesis Kit (Fermentas, Carlsbad, USA), and the reverse-transcribed products were used as the template for qRT-PCR with miRNA-specific primers (Additional file 1: Table S1). All reactions were assayed in three biological and technical replications and performed in an ABI-7300 (Applied Biosystems, Foster City, USA) using SYBR Green qPCR kit (Thermo Fisher, San Diego, USA). The relative expression level of miRNA was normalized to the internal control of *U6* small nuclear (*U6*). PCR conditions consisted of pre-denaturation and hot start Taq activation at 95 °C for 10 min, then 40 cycles of 95 °C for 15 s, and 60 °C for 45 s, and the relative expression was calculated by using the 2^-ΔΔCt^ method.

### miRNA target gene prediction, GO enrichment and KEGG pathway analysis

In the present study, the target genes of identified miRNAs were predicted using an *in silico* RNA-RNA hybridization approach following the protocol reported by Kruger & Rehmsmeier [32]. The program includes features such as: (i) perfect miRNA seed complementarity (positions 2 to 8) with 3'UTR sequence; (ii) *P*-value < 0.05; (iii) miRNA:mRNA binding energy < -20 kcal/mol; (iv) 1 maximum number of loops and internal bulges allowed; (v) no more than two adjacent mismatches in the miRNA/target duplex. 3'UTRs of *An. gambiae* downloaded from UTRdb [33] were used for the prediction. Mature miRNA sequence (fasta) and downloaded 3'UTR sequence was used as “query” and “target” files in RNAhybrid. The identities of predicted miRNA targets were determined by Kobas 3.0 [34] using *An. gambiae* as the reference species to limit annotations. Genes included in both the significant GO terms and KEGG pathways were further screened (enrichment score > 2 and *P* < 0.05). miRNA-mRNA network analysis was conducted for these selected targets and visualized by Cytoscape [35].

## Results

### Overview of *An. sinensis* sRNAs by sequencing

**Fig. 1:**
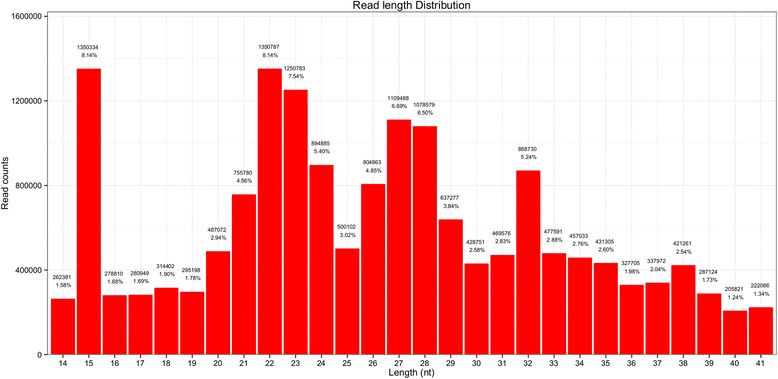
Length distribution for total sRNA reads of the *An. sinensis* library

We obtained a total of 24,502,168 raw sequence reads from a constructed small RNA library. After removing the low quality, adaptor sequences, and junk sequences, 16,586,625 high-quality sequence reads were retained for subsequent analysis (Table 1). Of these, 91.30% of the total reads and 85.55% of the unique reads mapped perfectly to the *An. sinensis* genome assembly. The length distributions of the total sRNA reads in the library are outlined in Fig. 1. The majority of sRNAs were between 19 and 24 nt, and the most abundant size class distribution was 22 nt, which accounted for 8.14% of the library, followed by 23 nt (7.54%), while the typical size range for Dicer-derived sRNA products are 20–24 nt. We removed a class of sRNAs about 24–27 nt in length (repeat-associated small interfering RNAs: rasiRNAs) which mediate the silencing of genomic repeats and transposons. We also removed sRNAs at 32–35 nt peak which represents longer piRNA-like small RNAs. The remaining reads were subsequently used for the identification of conserved miRNAs and the prediction of novel miRNAs.

**Table 1:** Categorization of reads of small RNAs in *An. sinensis*

Category	No. of reads	Percent (%)
Total reads	24,502,168	100
Reads trimmed adaptor	58,308	0.237970779
Reads trimmed N	41,501	0.169376849
Reads trimmed Quality Dynamics	11	4.4894E-05
Reads trimmed Quality Percent	1387	0.005660724
Reads trimmed PolyA/T	9973	0.040702521
Reads trimmed length	7,804,363	31.8517243
Clean reads	16,586,625	67.69451993

### Identification of conserved miRNAs and novel miRNAs by deep sequencing

To identify conserved miRNAs in *An. sinensis*, the remaining small RNA sequences were Blastn searched against miRBase v.21 [22]. A total of 73 miRNAs were identified in *An. sinensis*. Among them, 49 miRNAs were identified as conserved (irrespective of 5p and 3p), and these miRNAs belong to 46 different miRNA families (Additional file 2: Table S2). The top ten most abundant miRNAs are asi-miR-281, asi-miR-8, asi-miR-14, asi-miR-277, asi-miR-276, asi-miR-1891, asi-miR-10, asi-miR-184, asi-miR-9c, asi-miR-316 and asi-miR-275. After identifying the conserved miRNAs described above, the un-annotated sRNAs from the library were subjected to novel miRNAs prediction by searching against the *An. sinensis* genome. Moreover, we also considered the potential of miRNA precursors or sequences to form a hairpin structure. In total, 12 miRNA candidates were assigned as novel miRNAs as shown in Table 2. Novel miRNAs were tentatively classified into known miRNAs families by blasting against miRBase. However, they showed no conserved seed region with known miRNAs from other species. These miRNA sequences were designated in a specific order as detailed in Table 2.

**Table 2:** Details of predicted novel miRNAs by the deep sequencing

Mature name	Mature sequence	Precursor name	Precursor sequence	Strand	Precursor coordinate at genome
asi-miR-nov1	ugagaucaugacaguucaucg	asi-mir-nov1	ggugaaaugcuguguucucauaguaaacuacuggcuucuaugagaucaugacaguucaucg	+	gi|527265913|23455–23516
asi-miR-nov2	uaguacggugcgacuccccgu	asi-mir-nov2	cgggugagucuugccguacuacguguacuuuuguuauucucguaguacggugcgacuccccgu	+	gi|527266706|776475–776538
asi-miR-nov3	ugacuagauugcuuuggcuagu	asi-mir-nov3	cagcgaaagugguuuaguuuagcgcgcuuauucgaauguguugacuagauugcuuuggcuagu	+	gi|527270116|1744899–1744962
asi-miR-nov4	auuagaauguggaaucuguuuuu	asi-mir-nov4	auuagaauguggaaucuguuuuuguacguguuacagaaauaugcaaaaaaguuuucauauucuugcgg	-	gi|527266109|2021832–2021900
asi-miR-nov5	ucuaucauuugaguaccauga	asi-mir-nov5	cgugguacucuuuugguacggaguuucaaguaaagaauaccaucucuaucauuugaguaccauga	+	gi|527269397|156302–156367
asi-miR-nov6	ucaugucgacgcauccucugauu	asi-mir-nov6	aggaggauguguuggucaugaugguauuuuuucacaucaugucgacgcauccucugauu	-	gi|527231551|703–762
asi-miR-nov7	cggcccggaucguucgcaca	asi-mir-nov7	cggcccggaucguucgcacacgccagagcgaacgcauacgggcugcc	-	gi|527266065|40908–40955
asi-miR-nov8	gauucccucccuacuggacguacc	asi-mir-nov8	gauucccucccuacuggacguaccaaccguacagccggggucggggucuaauc	-	gi|527266109|1340028–1340081
asi-miR-nov9	gaggagcugcaggccgcc	asi-mir-nov9	cgcccgucgcccuucgucagccgguacgacuucaauggcgccgagguggacgaggagcugcaggccgcc	+	gi|527269794|806826–806895
asi-miR-nov10	aacgagcgucccggaccgcc	asi-mir-nov10	uggcccguaggugcuacguucguacgcguuacgaucgaacgagcgucccggaccgcc	-	gi|527266084|1600353–1600410
asi-miR-nov11	agcgggcucgagcggucacc	asi-mir-nov11	gacuguuccacccuccgucacaccaaagcaagcgggcucgagcggucacc	+	gi|527266274|736982–737032
asi-miR-nov12	ugcauucaguggggcggucgc	asi-mir-nov12	gauccuccuccguggauggcacguagucccaguugcuaaccggcgugcauucaguggggcggucgc	-	gi|527266901|716673–716739

### Overview of *An. sinensis* miRNAs by the bioinformatic approach

**Fig. 2:**
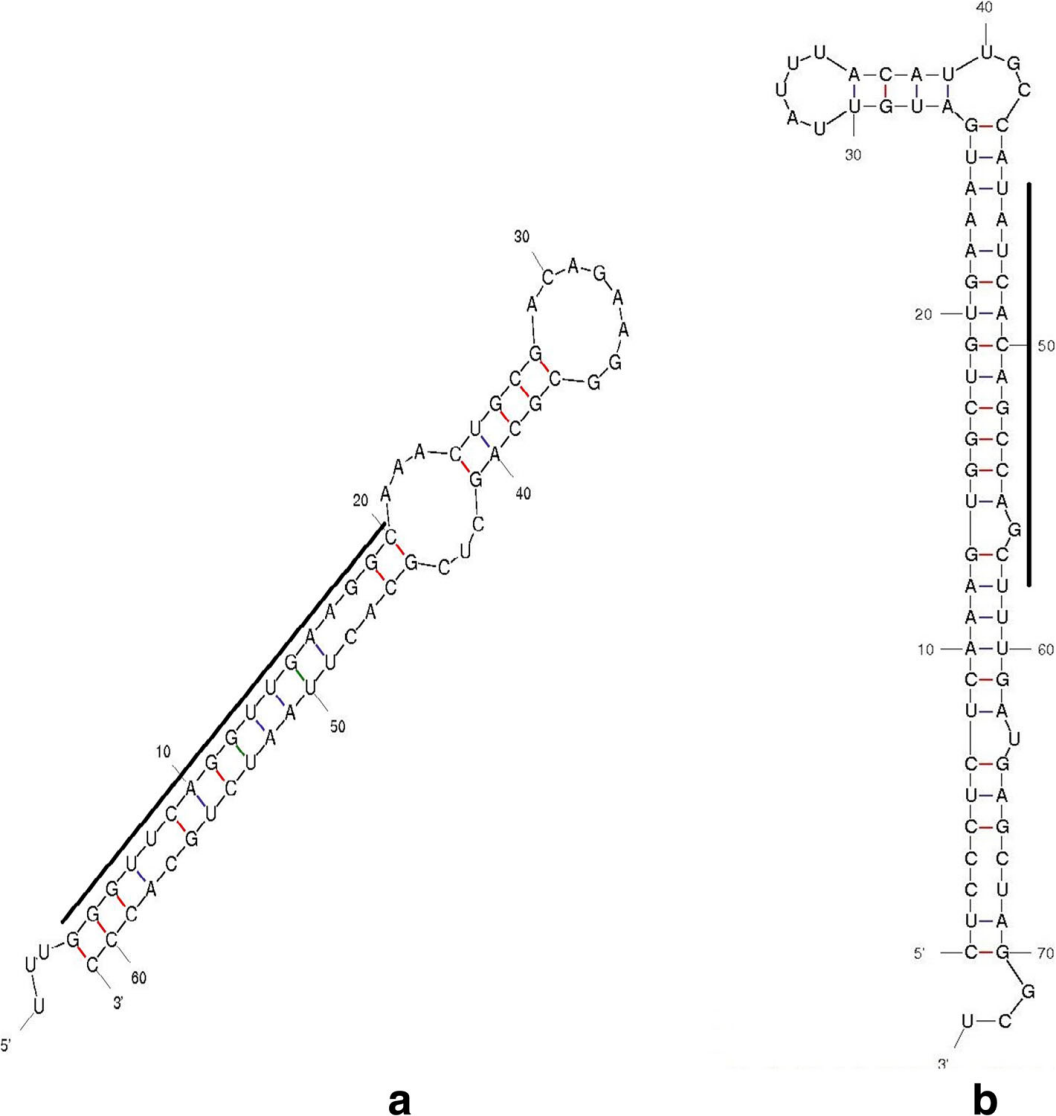
Precursor miRNA hairpin structures of *An. sinensis*, the underlined nucleotides indicate the mature miRNAs. **a** Precursor miRNA hairpin structures of asi-mir-9383. **b** Precursor miRNA hairpin structures of asi-mir-2a-2

Our pipeline yielded a number of putative miRNAs by nucleotide BLAST of 3119 stem-loop reference sequences resulting in 68 orthologous pre-miRNA signatures in 26 searched organisms of the subphylum Hexapoda. After removing the homologues, 43 miRNAs were identified in the *in silico* search (Additional file 3: Table S3). Among them, we have noted that 41 miRNAs have been deposited in Vectorbase, so only two *An. sinensis* miRNAs were reported as new after a careful evaluation of the secondary structure analysis (Fig. 2). These two miRNAs were noticed in plus strand and had the minimum free energy of -24.2 and -29.5 kcal/mol, respectively. Then, miRNAs were named according to the mirBase naming convention as asi-mir-9383 and asi-mir-2a-2 (as shown in Table 3). These two miRNAs identified in our study had homologues with mature miRNAs from insect species other than mosquitoes indicating that they may be species-specific.

**Table 3:** Details of predicted new miRNAs by the bioinformatic approach

Name	Source miRNA	Source organism	Mature sequence	Strand	Minimum free energy values (kcal/mol)	Coordinates in genome
asi-miR-9383	dme-miR-9383	*Drosophila melanogaster*	GGGUUCAGGUUGAAGGCAAACU	5'	-24.2	KE525292.1:9200–9260
asi-miR-2a-2	dme-miR-2a-2	*Drosophila melanogaster*	UAUCACAGCCAGCUUUGAUGAGC	5'	-29.5	KE525350.1:1783756–1783828

### Validation of predicted miRNA by qRT-PCR

**Fig. 3:**
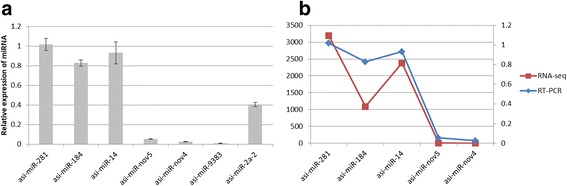
Validation of the selected known and novel miRNAs by quantitative real-time PCR. **a** Seven miRNAs (five miRNAs from the sequencing result and two miRNAs from the bioinformatic approach) were identified by qRT-PCR. **b** The relative expression of five miRNAs from the sequencing result showed similar expression pattern when confirmed by qRT-PCR. The transcript levels of both known and novel miRNAs were calculated relative to the amount of *U*6 small nuclear after normalization. The real time PCR data with bars represent the mean ± SD from three independent experiments

To identify the presence and the relative expression of the predicted miRNA, we selected known miRNAs and novel miRNAs from both the deep sequencing and the bioinformatic approach to perform qRT-PCR (Fig. 3a). All miRNAs (asi-miR-281, asi-miR-184, asi-miR-14, asi-miR-nov5, asi-miR-nov4, asi-miR-9383 and asi-miR-2a) showed in the sample. We also observed a similar trend for the expression patterns of these miRNAs measured by qRT-PCR when compared with that in the original miRNA profiling data (Fig. 3b), which in turn confirmed the sequencing result.

### Comparative analysis of miRNA profiles by the deep sequencing and the bioinformatic approach

**Fig 4:**
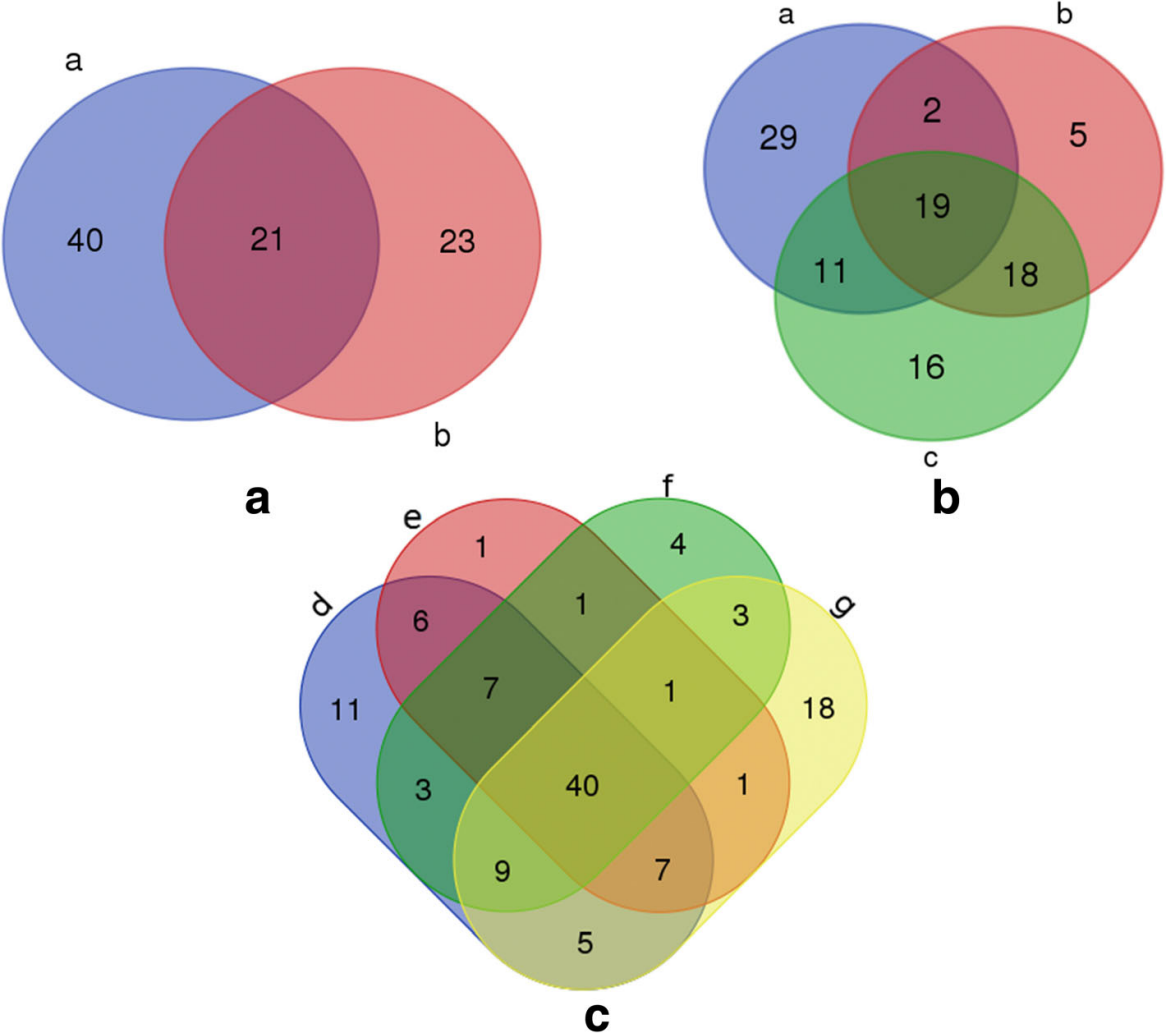
**a** Venn diagrams of the number of miRNAs predicted by deep sequencing and bioinformatic approach in *An. sinensis* (a, miRNAs predicted by deep sequencing; b, miRNAs predicted by bioinformatic approach). **b** Venn diagrams of the number of miRNAs predicted by deep sequencing, bioinformatic approach and miRNAs genes from genome in *An. sinensis* (a, miRNAs predicted by deep sequencing; b, miRNAs predicted by bioinformatic approach; c, miRNAs from genome prediction). **c** Venn diagrams of miRNAs for *Ae. aegypti*, *An. gambiae*, *Cx. quinquefasciatus* and *An. sinensis* from this study (d, mature miRNAs of *Ae. aegypti*; e, mature miRNAs of *An. gambiae*; f, mature miRNAs of *Cx. quinquefasciatus*; g, mature miRNAs of *An. sinensis* from this study)

Analysis of the miRNAs profiles by the deep sequencing and the bioinformatic approach is shown in Fig. 4a. A total of 84 miRNAs were identified by combining two approaches, among them, 21 miRNAs were shared between them. As for the 14 novel miRNAs in this study, two miRNAs were previously reported for *Drosophila melanogaster* and 12 did not match any known miRNAs in any organism. Compared with miRNA genes predicted from the genome of *An. sinensis* by Dritsou et al. [36] (Additional file 4: Table S4), 19 miRNAs (asi-miR-252, asi-miR-10, asi-miR-927, asi-miR-8, asi-miR-996, asi-miR-1, asi-miR-276, asi-miR-305, asi-miR-277, asi-let-7, asi-miR-283, asi-miR-210, asi-miR-133, asi-miR-988, asi-miR-275, asi-miR-315, asi-miR-14, asi-miR-31 and asi-miR-999) in the *An. sinensis* were shared within three databases as shown in Fig. 4b. Moreover, we also compared the miRNA profile of *An. sinensis* with miRNAs for *An. gambiae*, *Ae. aegypti* and *Cx quinquefasciatus*. The result showed that there is a high consistency (40 miRNAs) as far as total numbers of miRNAs identified in these taxa as shown in Fig. 4c*.*

### Novel miRNA target gene prediction, GO enrichment and KEGG pathway analysis

**Fig. 5:**
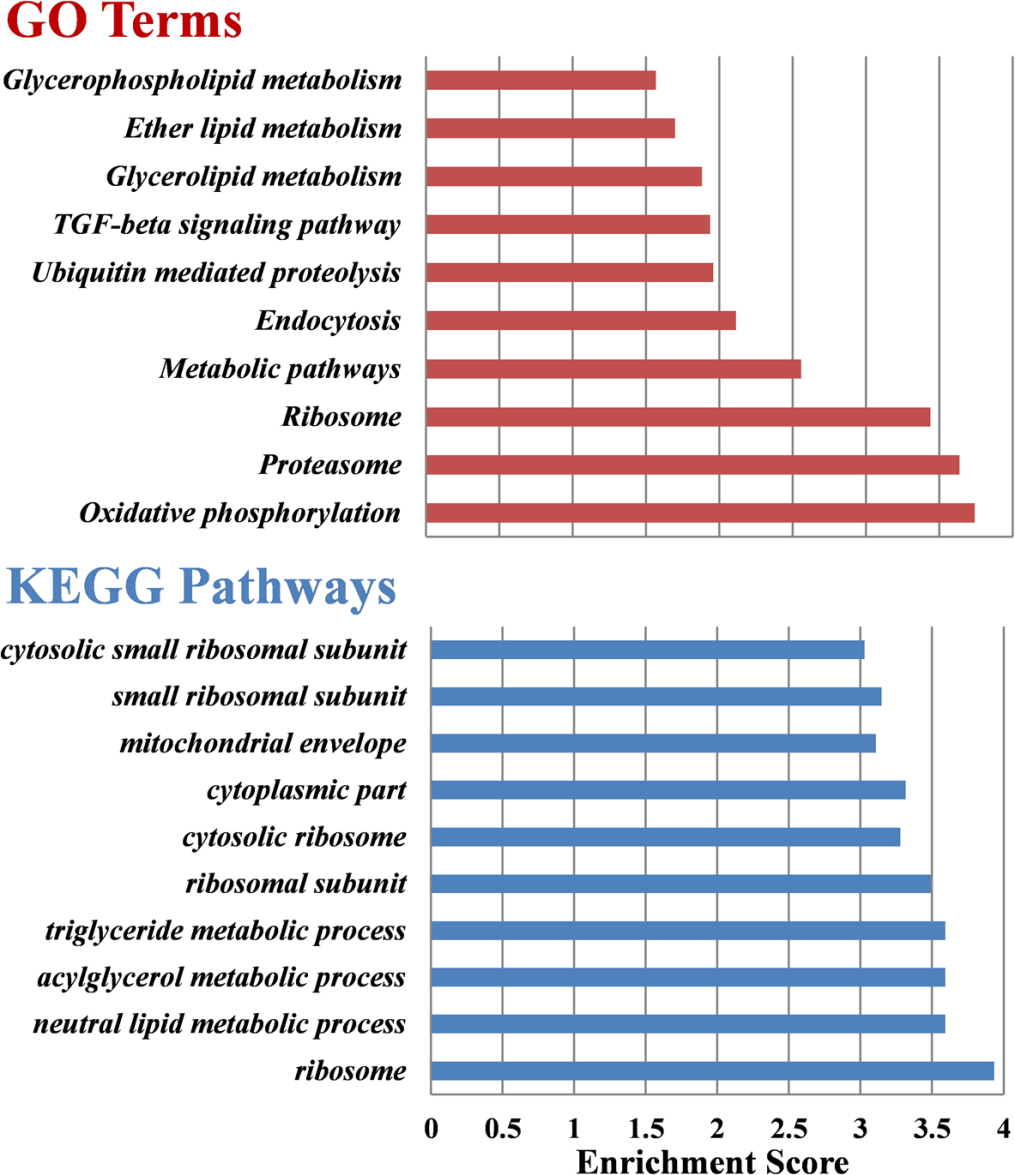
KOBAS analysis of miRNA targets predicted by RNA hybrid. Bar charts represent top 10 Gene Ontology terms and KEGG pathways targeted by miRNAs. Enrichment score was calculated by -log10 (*P* < 0.05)

The putative targets for the 14 novel miRNAs were performed using RNAHybrid to understand the putative biological roles. A total of 938 potential targets that satisfied the preset values were predicted (Additional file 5: Table S5). Maximum and minimum numbers of mRNA targets were predicted for asi-miR-nov11 (*n* = 173) and asi-miR-nov1/asi-miR-2a-2 (*n*  = 20), respectively. The targets were further analyzed by KOBAS. The predicted targets were found to be involved in a broad range of biological processes. The top 10 significant GO terms and KEGG pathways for the 14 novel miRNAs and putative target genes, respectively, are shown in Fig. 5. The most significantly enriched GO terms included ribosome (GO: 0005840), neutral lipid metabolic process (GO: 0006638), acylglycerol metabolic process (GO: 0006639), triglyceride metabolic process (GO: 0006641), ribosomal subunit (GO: 0044391), cytoplasmic part (GO: 0044444) and mitochondrial envelope (GO: 0005740). The most significantly enriched KEGG pathways included pathways in oxidative phosphorylation (aga00190), proteasome (aga03050), ribosome (aga03010), metabolic pathways (aga01100) and endocytosis (aga04144). asi-miR-nov9 and asi-miR-nov8 were identified to target oxidative phosphorylation and metabolic pathway, and miR-nov5 was also found to target proteasome and endocytosis. In addition, miRNA:mRNA interaction was analyzed through network generation as shown in Additional file 6: Figure S1. We identified 105 transcripts that were targeted by two or more than two miRNAs. Fifty transcripts (such as AGAP000951, AGAP003493, AGAP003720, AGAP004400, AGAP004880 and AGAP008327) were targeted by miRNAs in different transcript regions. Some of these are of great importance during the developmental signaling pathway and immune pathway. For example, AGAP003493 is one member of sugar transporter which facilitates the transport across cytoplasmic or internal membranes of a variety of substrates [37]. Also, AGAP008327 has a significant differential expression in the female salivary gland and 1.4-fold up-regulation with respect to high-intensity *Plasmodium berghei* infection [38].

### Conserved miRNA in *An. sinensis* and its functions

Previous evidence showed that miRNAs could negatively regulate the immune response in *An. gambiae* [18, 39]. Similarly, we identified the published conserved orthologues of miRNAs and multiple corresponding target transcripts of the immune pathway in *An. gambiae*. In total, we identified 21 conserved miRNAs and 8 mRNA counterparts in *An. sinensis* (Additional file 7: Figure S2). *PGRP* (peptidoglycan recognition protein), Caspar, and *Rel2* (*NF-κB* transcription factor) have been reported to play crucial roles in the immune signaling pathway [40]. Further analysis by miRNA-mRNA interaction network showed that asi-miR-275 and asi-miR-305 contained predicted binding sites within the 3'UTR of *PGRP-LB* and *APL1C*, respectively, and the 3'-UTR of the anti-*Plasmodium* genes, such as *PGRP-LC*, *PGRP-LD*, *Rel*2, *Caspar*, *IMD* (immune deficiency pathway) and *LRRD*7*/APL*2, had *in silico*-predicted binding sites of many miRNAs. For instance, *PGRP-LD* had the potential to interact with asi-miR-100, asi-miR-124, asi-miR-210, asi-miR-278, asi-miR-282, and asi-miR-989. The asi-mir-2 was found to interact with the three key immune genes in IMD pathway that lead to an increased expression of antimicrobial peptides [18]. These results indicate that interaction of miRNAs with their targets could lead to regulation of immune proteins which further mediate midgut infection in *An. sinensis*.

## Discussion

miRNAs have been discovered in many mosquito species, including *An. gambiae*, *Ae. aegypti* and *Cx. fatigans* [14, 16–18, 41]. In the present study, the miRNA expression profile of *An. sinensis* was analyzed by NGS technology and compared with the predicted results of the bioinformatic approach. From the *An. sinensis* small RNA data set, we identified a total of 49 conserved miRNAs which belong to 46 miRNA families, and 12 non-conserved (novel) miRNAs. This indicates that the number of conserved miRNAs is probably much larger than that of novel miRNAs in *An. sinensis*. In addition, after a careful evaluation of the secondary structure analysis, another 42 conserved and two novel miRNAs were yielded by the bioinformatic approach for orthologous signatures in 26 organisms of the subphylum Hexapoda. In total, 14 miRNAs were assigned as the novel in this study. Among them, 12 miRNAs lack orthologues with recorded species in Vectorbase, indicating that these miRNAs could be unique for *An. sinensis*.

The number of miRNAs determined through our pipeline is almost consistent with what has been predicted earlier for other mosquito species, such as 65 miRNAs for *An. gambiae*, 124 miRNAs for *Ae. aegypti*, 93 miRNAs for *Cx. quinquefasciatus* [42]. More interestingly, 40 miRNAs were found to be shared by all three taxa analyzed above. Of those, some conserved miRNAs have been found in a large variety of insect species, which indicate a highly conserved structure or role in evolution. In our study, the most abundantly expressed miRNAs, such as miR-8, miR-10, miR-184 and miR-281, exhibit a comparable expression pattern in *An. gambiae* [43]*.* In addition, some miRNAs, for instance, miR-184 was also reported as the most frequently occurring miRNA in other mosquito species [15, 44]. In brief, comparisons between miRNA profiles by two approaches revealed the fundamental miRNA profile in Chinese malaria vector *An. sinensis*. However, the temporal and spatial expression patterns and related functions, still need to be further validated through biological experiments.

To validate our sequencing and the bioinformatic approach results, we performed qRT-PCR analysis using the RNA sample used for small RNA NGS. Eight miRNAs from our sequencing results and novel miRNAs from the bioinformatic approach were selected for validation. All selected miRNAs (asi-miR-281, asi-miR-184, asi-miR-14, asi-miR-nov5, asi-miR-nov4, asi-miR-9383 and asi-miR-2a) showed in the sequencing sample. Five miRNAs showed similar expression patterns as those revealed by our sequencing analysis, which indicated a low false discovery rate of our sequencing data and supported the validity of the profiling. Two selected miRNAs (asi-miR-281, asi-miR-184, asi-miR-14, asi-miR-nov5, asi-miR-nov4, asi-miR-9383 and asi-miR-2a) from the bioinformatic approach result could also be confirmed by quantitative real-time PCR, indicating that we may have omitted some novel miRNA prediction during analyzing sequencing results.

The majority of known mosquito miRNAs have been determined through traditional direct cloning [45], bioinformatic prediction [16, 28] or deep sequencing [15, 46]. In the present study, both bioinformatic prediction and the deep sequencing were applied. Next-generation sequencing (NGS) has become an innovative tool with the unprecedented depth of coverage to uncover miRNAs in many species [47]. This new approach has the ability to find both old and new miRNAs profiles by high throughput and fine resolution at a single base. However, sequences with low abundance may not be detected and predictive ability may be restricted if there is no reference genome [48]. Besides, the bioinformatic prediction is also widely used for the identification of miRNAs depending on similarity searches or algorithms based on evolutionary conservation [49]. However, it may miss species-specific and rapidly evolved miRNAs. In addition, miRNAs have significant variation in their expression levels in different time scales and tissues or under different conditions. This also makes it difficult to identify miRNAs by a single method [8]. Thus, as an alternative, joint usage of NGS and bioinformatics technology by taking advantage of their own strengths would undoubtedly facilitate the discrimination of vectors’ miRNA profiles.

To understand various biological processes of miRNAs, it is necessary to study their potential targets. A total of 938 potential targets were predicted for the 14 novel miRNAs. All predicted targets have crucial biological roles ranging from metabolic process to mitochondrial envelope which might be important for development. Some KEGG pathways related to oxidative phosphorylation, proteasome and ribosome were also identified. More than one hundred transcripts were targeted by two or more miRNAs. Meanwhile, 50 transcripts were regulated by miRNAs in different regions, suggesting that miRNAs are involved in complicated regulatory networks as suggested by other studies [8, 36, 50]. The conserved miRNAs in *An. sinensis* were found to have *in silico*-predicted binding sites within many transcripts as described before in *An. gambiae* [18]. Significantly, these conserved orthologues play important roles in regulating pathogen infection [39]. Although, miRNA target genes are commonly recognized and bound by the miRNA seed region at the 3'-UTR, the recently identified miRNA binding sites within the 5'-UTR as well as the coding regions of target genes yield more target genes for interest miRNAs [9]. Deciphering the function of these cleaved targets identified in *An. sinensis* would provide us with a better idea about their role in both mosquito biology and pathogen infection.

## Conclusions

In conclusion, our study provides a comprehensive account of the miRNA profile of *An. sinensis* by combining the deep sequencing and the bioinformatic approach. Efforts were undertaken to understand the targets of miRNAs which can provide a better understanding of their biological function in mosquito biology and immunity and provide implications for effective control in the future. However, further experimental studies will be needed to validate these predictions and their interactions.

## Additional files

Additional file 1: **Table S1.** miRNA-specific primers used in qRT-PCR validation. (XLSX 10 kb)

Additional file 2: **Table S2.** miRNAs predicted from small RNA sequencing data of *An. sinensis*. (XLSX 33 kb)

Additional file 3: **Table S3.** miRNAs predicted by the bioinformatic approach. (XLSX 141 kb)

Additional file 4: **Table S4.** miRNA genes predicted from the genome of *An. sinensis*. (XLSX 17 kb)

Additional file 5: **Table S5.** List of miRNA targets identified by RNAhybrid on *An. sinensis* genes. (XLSX 35 kb)

Additional file 6: **Figure S1.** miRNA:mRNA interaction network of novel miRNAs and their predicted targets. Interaction network of novel miRNAs and their targets. miRNAs are diamond-shaped whereas targets are elliptic. Transcripts targeted by two or more than two miRNAs are marked in yellow color. (PDF 64 kb)

Additional file 7: **Figure S2.** miRNA:mRNA interaction network of conserved miRNAs and their predicted targets in the immune pathway. (TIFF 897 kb)

## Abbreviations

GO: Gene ontology
IMD: Immune deficiency pathway
KEGG: Kyoto Encyclopedia of Genes and Genomes
miRNAs: microRNAs
NGS: Next-generation sequencing
PGRP: Peptidoglycan recognition protein
qRT-PCR: quantitative reverse transcription real-time polymerase chain reaction
sRNAs: small RNAs
UTR: Untranslated regions
