# Supplementary figures and images for: Analysis of microRNA profile of Anopheles sinensis by deep sequencing and bioinformatic approaches
Source: Parasit Vectors. 2018 Mar 12;11:172. doi: 10.1186/s13071-018-2734-7 (PMC5848538; doi:10.1186/s13071-018-2734-7)

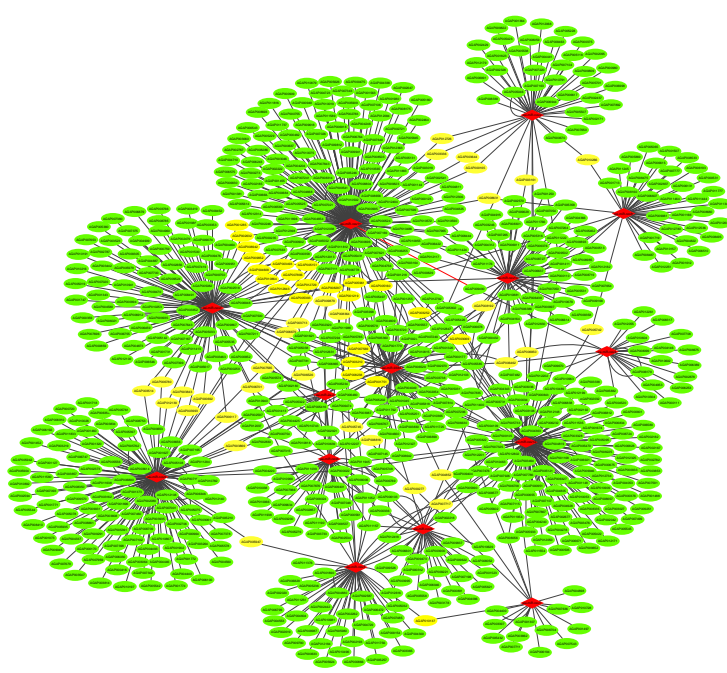

Supplement: Supplementary file 6 — Figure S1. miRNA:mRNA interaction network of novel miRNAs and their predicted targets. Interaction network of novel miRNAs and their targets. miRNAs are diamond-shaped whereas targets are elliptic. Transcripts targeted by two or more than two miRNAs are marked in yellow color. (PDF 64 kb) [file 13071_2018_2734_MOESM6_ESM.pdf]

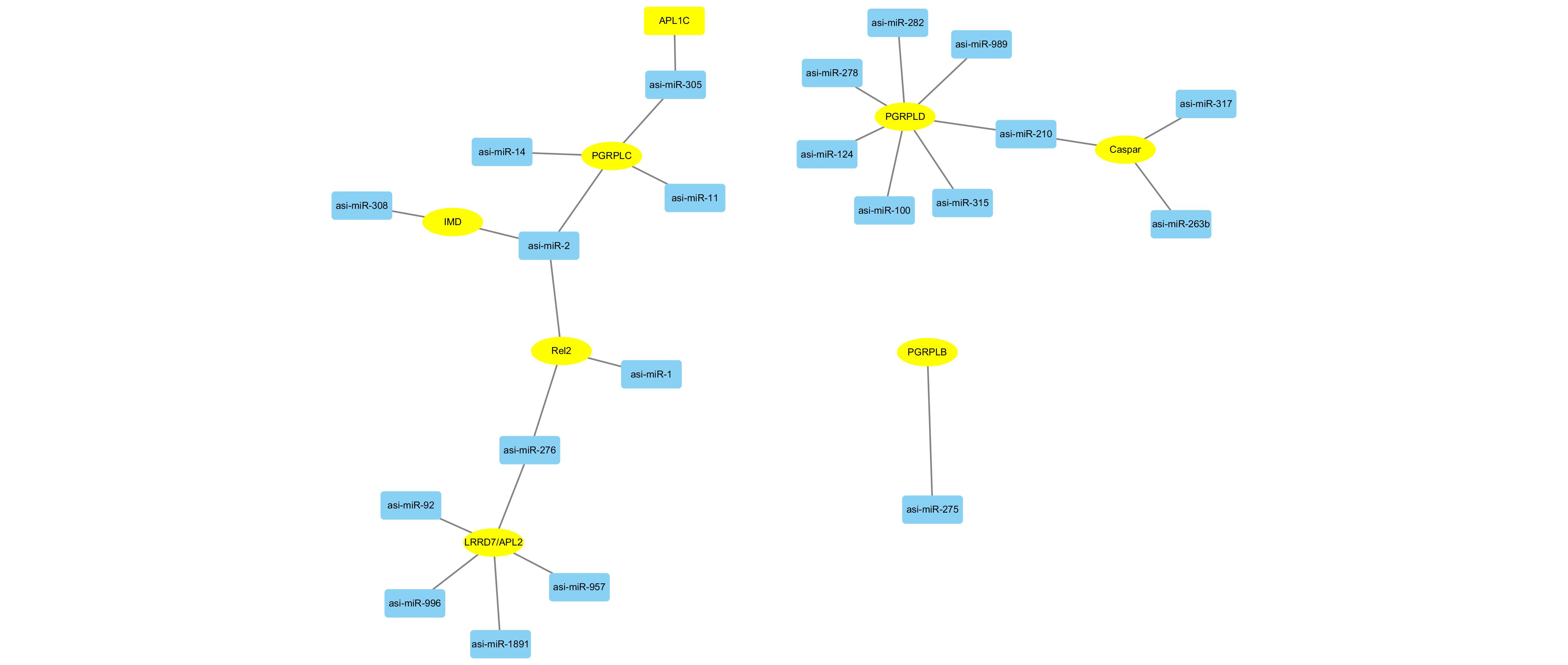

Supplement: Supplementary file 7 — Figure S2. miRNA:mRNA interaction network of conserved miRNAs and their predicted targets in the immune pathway. (TIFF 897 kb) [file 13071_2018_2734_MOESM7_ESM.tif]
